# Post-Traumatic Growth in Women with Breast Cancer: Intensity and Predictors

**DOI:** 10.3390/ijerph19116509

**Published:** 2022-05-27

**Authors:** Justyna Michalczyk, Joanna Dmochowska, Anna Aftyka, Joanna Milanowska

**Affiliations:** 1 Student Scientific Association, Chair and Department of Psychology, Medical University of Lublin, ul. Chodźki 7, 20-093 Lublin, Poland; joannadmochowska.um@gmail.com; 2 Department of Anaesthesiological and Intensive Care Nursing, Medical University of Lublin, ul. Chodźki 7, 20-093 Lublin, Poland; anna.aftyka@umlub.pl; 3 Chair and Department of Psychology, Medical University of Lublin, ul. Chodźki 7, 20-093 Lublin, Poland; joanna.milanowska@umlub.pl

**Keywords:** PTG, post-traumatic growth, breast cancer, resilience, trauma

## Abstract

Experiencing a traumatic situation such as breast cancer can, beside negative consequences, have a positive impact, described as post-traumatic growth (PTG). A factor that facilitates psychological recovery when coping with stressful events is psychological resilience. The aim of the present study was to assess whether PTG occurs in a group of women with breast cancer and whether resilience is a personal trait contributing to its occurrence. The study group comprised 100 women with breast cancer, aged 31–80 years, almost half of whom were aged 61–70 years (*n* = 46, 46%). The Post-Traumatic Growth Inventory, the Impact of Event Scale, and the Resilience Assessment Questionnaire (KOP) were used for the study. All women manifested PTG, with a mean intensity of 76.61 ± 13.45 points. The greatest changes were observed in the subjects’ appreciation of life, and the smallest in their relations with others, self-perception, and spiritual changes. The KOP scale measured a mean resilience of 103.80 ± 16.57. The results obtained confirm the co-occurrence of psychological resilience and PTG, especially personal resilience and social competences. Additionally, women subjected to additional traumatic events other than cancer manifested a higher level of PTG.

## 1. Introduction

Cancer is a genetic disease caused by a DNA mutation. Epidemiological data show that cancer is the second most common cause of death in Poland [1]. Worldwide, 1.7 million women develop cancer each year, and in 2018 the mortality rate was 678 thousand [2].

Rehabilitation involving physical exercises and psychological help is an important element of cancer treatment. The goal of psycho-physical rehabilitation is to reduce the effects of physical treatment (e.g., lymphoedema, the swelling of the upper limb on the operated side) and also to mitigate the negative psychological effects, for example, social and occupational maladjustment, family issues, and the fear of mutilation, suffering, or death [3]. Psychological therapy also aims to reduce stress and restore a patient’s emotional state to the time before the disease. Promoting positive emotions and reinforcing the strengths create opportunities for patients to improve the quality of their lives [4].

Cancer treatment is a long-lasting and exhausting experience, both for the body and mind of the patient. Breast amputation in particular may be a traumatic experience, often leading to a loss of self-worth. Proper medication, including chemotherapy, causes hair loss and a general worsening of one’s appearance. Therapeutic success is hugely affected by support from family, friends, psychologists, and psychiatrists. Many women with breast cancer seek support from organisations uniting women with various breast cancers. Throughout Poland, over 70 such associations, known as “Amazons Clubs”, aim to educate, run social campaigns, and lend ongoing support to people struggling with cancer [5].

Cancer is a traumatic experience for the patient, affecting their functioning in many respects. Trauma is defined as a psychological injury brought about by strong emotional stimuli. The concepts of trauma and stress are related, but clinical psychology makes a clear distinction between trauma and a stressful situation. Stressful events are those a person can cope with. In the case of trauma, coping with adversity is very difficult or impossible. Currently, the World Health Organization defines traumatic events as exposure to a severely threatening or catastrophic short- or long-term stressor. When describing trauma, emphasis is placed on the affected person’s self-appraisal [6].

In some people, traumatic situations can bring about not only negative consequences but also positive changes, described as post-traumatic growth (PTG) [7]. The occurrence of trauma does not automatically lead to growth; it is a process that involves a strategy in dealing with a dramatic experience. The sense of personal happiness, for example, having overcome an illness, cannot be identified as PTG. Research has shown that PTG occurred in 30–90% of those who had been through traumatic situations [8].

The term “post-traumatic growth” was first used in 1996 by Tedeschi and Calhoun [9]. They defined post-traumatic growth as “positive change that the individual experiences as a result of the struggle with a traumatic event” [10]. Later, they further specified that PTG was “a struggle with the new reality in the aftermath of trauma that is crucial in determining the extent to which post-traumatic growth occurs”. Positive changes include better self-perception, improved interpersonal relationships, and a change in life philosophy [11]. This may manifest as greater resistance to stress, taking up various life challenges, and greater appreciation of life and loved ones, as well as changes in religious spheres [9]. Regarding the conceptual model of post-traumatic growth, it has been defined as an innate biological mechanism whose role is to protect the person against the effects of stress [12].

Trauma can be treated as a basis for personal growth. Nonetheless, its occurrence does not determine that the negative effects of cancer will be experienced as, for example, PTSD. People affected by PTG can manifest distress and low well-being [13].

Good social relations, marriage and good family relationships, younger age, and a shorter diagnosis time in the case of cancer all favour growth [13]. Authors point to the positive influence of religious beliefs on PTG, viewing increased coping with stress, forgiveness, and sense of belonging as positive factors [14].

It has been found that PTG in oncological patients was greatly affected by traits such as the ability to adapt to a difficult situation, good mental and physical health, slight symptoms of distress and post-traumatic stress, and rigid discipline during treatment [4]. Scientific research has demonstrated that, in the case of breast cancer, PTG is influenced by factors such as awareness of the invasive impact of the disease on the body, relapse concerns, perception of cancer as a life-threatening disease, and the intensity of the anti-cancer therapy. It follows that in women who have survived breast cancer, PTG is closely linked to coping based on emotions, support of others, problem coping, and adaptation to new life circumstances [15].

One dimension that can determine a person’s adaptive capabilities when faced with a traumatic situation, and subsequently affect PTG, is resilience. The word “resilience” comes from the Latin ”salire’ and means ”spring, spring up”. “*Resilire*”, therefore, means ”spring back”, or go back to the original state. Resilience is understood as psychological endurance, which can be interpreted as a personality trait manifesting as the ability to cope in a particular situation, a process, or its result [16].

Broadly speaking, resilience is a trait that enables a person to function optimally despite failures. It is an ability to stay away from negative experiences; it is a type of positive adaptation associated with trauma [17]. Experts underscore the significance of developing resilience during a lifetime and point to the importance of positive social factors in this process. This competence is essential in overcoming difficulties in life, stress, or traumas [18].

The aim of the present study is to establish the level of PTG within a group of cancer patients, as well as the relationship between PTG and sociodemographic variables such as age, time from diagnosis, relationship status, survival of additional traumatic events, and resilience. The results obtained in the study should be of help to psychologists and doctors, as well as others who want to support women struggling with cancer, such as family members.

To date, research on PTG has not focused on patients associated with Amazon Clubs in Poland. Due to cultural differences, it seemed reasonable to recruit Polish women with breast cancer for this research.

## 2. Materials and Methods

This study is a cross-sectional study and is based on a questionnaire. We surveyed 100 Amazon Club members throughout Poland, all of whom had breast cancer in the past. When starting the study, the maximum number of participants was not specified. An electronic version of the questionnaire was made available and 150 paper-based research questionnaires were sent to Amazon Clubs all over Poland. Not all questionnaires were completed despite participants’ prior declarations of intent. Some of the questionnaires were filled in incorrectly and had to be rejected. In total, we received 115 questionnaires, 100 of which were completed correctly. Consent was obtained from the Bioethical Commission (KE-0254/165/2021) to conduct the research. The respondents were informed about their anonymous status and the purpose of the survey.

The following tools were used:

**Post-Traumatic Growth Inventory (PTGI)** This tool was authored by Tedeschi and Calhoun, adapted for the Polish context by Ogińska-Bulik and Juczyński. It consists of 21 statements concerning positive changes resulting from a negative life event. Each surveyed person responds to the statements by choosing what they perceive to be the most appropriate answer, from “*I did not experience this change*” (0 pts) to “*I experienced this change to a very great degree*” (5 pts). Choosing a higher score testifies to a greater intensity of the positive change. The inventory comprises four subscales: (1) Changes in self-perception, (2) changes in relating with others, (3) greater appreciation of life, and (4) spiritual change [8].

**Impact of Event Scale—Revised** This tool was developed by Weiss and Marmur, and adapted by Juczyński and Ogińska-Bulik. The 22 statements measure post-traumatic stress disorder (PTSD), with each statement relating to a specific symptom. Respondents mark their answers on a 5-point scale (from 0 to 4). The total score enables a screening assessment of post-traumatic stress. The scale also makes it possible to assess the chief symptoms of PTSD: (1) Intrusion, (2) arousal, and (3) avoidance [19].

**KOP-26** This tool was developed by Gąsior, Chodkiewicz, and Cechowski. It examines resilience, defined as personal, family, and social competences, allowing people to better cope with traumatic events, stress, and life problems. It comprises 26 statements, assessed on a 5-point scale. The personal competences examined are related to the ability to pursue targeted and responsible action, as well as to maintain the sense of the meaning of one’s life. Family competences are regarded as mutual understanding, a sense of support from loved ones, and friendly family atmosphere. Social competences are related to the ability to find oneself in a new situation, and openness to being helped by others [20].

## 3. Results

### 3.1. Characteristics of the Study Group

In the study group (*n* = 100), almost half of the respondents were women aged 61–70 years (*n* = 46, 46%), and the large majority lived in a town (*n* = 91, 91%). The majority of respondents had lived with the diagnosis for over 10 years (*n* = 60, 60%), putting patients who had lived with their diagnosis for 5–10 years (*n* = 18, 18%) and less than 5 years (*n* = 22, 22%) in the minority. Almost half of respondents were college educated (*n* = 49, 49%), and three-fifths of the respondents were married (*n* = 60, 60%). Approximately one-third of respondents (*n* = 35, 35%) reported traumatic events other than cancer, and three-quarters of the surveyed presented symptoms suggestive of PTSD. See Table 1 for a study group description.

### 3.2. Results

The mean level of PTG measured using the PTGI in the study group was 76.61 ± 13.45 points. The respondents were the most likely to perceive changes in their appreciation of life (4.00 ± 0.86 pts), but less likely to perceive changes in their appreciation of relating with others (3.73), self-perception (3.60 ± 0.73), and spiritual change (2.95 ± 1.33). The mean PTSD level assessed using the IES-R was 49.57 ± 16.08 points. The strongest PTSD symptoms were intrusion (2.33 ± 0.85) and arousal (2.22 ± 0.79); avoidance was weaker (2.19 ± 0.72). The mean resilience assessed using the KOP scale was 103.80 ± 16.57. The descriptive statistics of the quantitative variables are presented in Table 2.

A correlation was found between PTG and resilience (r = 0.54, *p* < 0.05), especially in two resilience subscales: personal resilience (r = 0.54, *p* < 0.05), and social competences (r = 0.45, *p* < 0.05). A weaker correlation occurred between post-traumatic growth and family competences (r = 0.38, *p* < 0.05), arousal (r = 0.31, *p* < 0.05), and overall severity of post-traumatic stress symptoms (PTSS; r = 0.30, *p* < 0.05). The correlation matrix is presented in Table 3.

No statistically significant differences in PTG and its components were found in women of different ages (see Table 4), in women with illnesses of different durations (see Table 5), or in married and unmarried women (see Table 6).

Women who stated living with cancer was their only experience of a traumatic event presented a lower PTG level as compared with women who had experienced many traumatic events (74.46 ± 13.30 and 80.60 ± 12.98, respectively, Z = 2.38, *p* < 0.05). Women who had experienced only one traumatic event (cancer) also manifested lower changes in self-perception and relating with others as compared with those who had experienced multiple traumatic events (see Table 7).

## 4. Discussion

### 4.1. The Level of Post-Traumatic Growth

Our aim was to demonstrate a relationship between PTG and factors such as spirituality, appreciation of life, self-perception, and relating with others.

Psychological theory identifies PTSD and PTG as separate phenomena, related to each other in complex ways. In this relationship, stress and suffering are a catalyst triggering growth. The causes of PTG are rooted in changes in fundamental beliefs about oneself and the surrounding world, which arise when an individual is confronted with extreme events [21]. Research, to date, indicates that the trauma associated with the diagnosis, treatment, and overcoming of breast cancer leads to PTG in some patients. A direct confrontation with death activates stress and triggers mechanisms generating PTG. Suffering is transformed into a value that is beneficial to the sufferer. Based on the available research on PTG in persons who have grappled with cancer, we can conclude that PTG is the prevailing tendency among respondents [22].

The present PTGI-based study demonstrates a mean growth of PTG by 76.61 ± 13.45 points, which seems to corroborate the present theoretical model of PTG in an oncological disease. This result shows that traumatic events related to breast cancer prompt growth in patients. It is adaptive and results from cognitive processing of traumatic events. A systematic review of 47 papers has shown that breast cancer patients who have undergone chemotherapy or radiotherapy manifest growth which is positively correlated with appreciation of life following dramatic experiences, changes in self-perception, and relating with others [23]. The data we collected suggest that women with breast cancer noticed positive changes in their appreciation of life (4.00 ± 0.86 points). Slightly lower growth occurred in relating with others (3.73) and self-perception (3.60 ± 0.73).

Researchers in China split a group of 612 women who previously had breast cancer into three groups based on the relationship between PTG incidence and the intensity of PTSS. Three types of patterns were isolated: a growth group (mild PTSS, high PTG) accounting for 47.4%, a resilient group (mild PTSS, mild PTG) at 34.6%, and a struggling group (high PTSS, high PTG) constituting 18%. No group was identified among the Chinese respondents that manifested severe PTSS and no growth, which was consistent with studies conducted in other cultures. It was also noted that various kinds of traumatic events generated different levels of PTG [23].

### 4.2. Relationship of PTG with Sociodemographic Variables

It can be concluded that mechanisms of PTG formation depend not only on individual predisposition, but also on general social and cultural conditions. Cross-sectional analyses conducted in recent years to examine the relationship between suffering and growth in women with breast cancer have indicated that the occurrence of PTG was accompanied by stress alleviation and improvement in the psychological condition of the respondents. Conversely, women with low PTG manifested increased depressive symptoms and lower mood. A 2017 study conducted using the PTGI confirmed that much lower PTG was evident in depressed women, particularly in areas such as life evaluation and seeing new opportunities [24].

Research was conducted in Iran on a group of 210 women with diagnosed breast cancer and showed that the satisfaction of basic needs was positively related to PTG. It was assumed that individuals have a better chance of coping with a traumatic situation such as an oncological illness if their basic needs, such as autonomy, competence, or family ties, have been met. They treat such a tough experience as a confrontation with reality and they have a sense of control over events [25].

Similar research has been conducted, in 2019 in Croatia, that demonstrated a positive link between PTG and sense in life, seeking the meaning of life and life satisfaction. The sample consisted of 149 patients who had recovered from cancers, affiliated with an oncological organisation. The respondents manifested a mild degree of PTS and positive changes (*M* = 3.33, *SD* = 1.15; total PTG = 62.68, *SD* = 22.68). PTG was at least at a medium level in 72.5% of the participants. Interestingly, the analysis also showed that PTG was dependent on cancer location. Breast cancer survivors manifested higher PTG than those who had recovered from mouth, throat, or larynx cancer [26]. A longitudinal study conducted over 8 months from diagnosing first-to-third-degree breast cancer in 653 female patients at the Memorial Sloan Kettering Cancer Center and the University of Texas Southwestern Center for Breast Care confirmed that PTG developed over time, and that most patients observed the changes within the first year of the diagnosis. In this study, higher PTG was positively linked to education, longer time elapsed since diagnosis, higher initial level of cancer intrusion, and the growth of social support [27].

Over time, cancer survivors experience growth in such areas as approach to life and relating with others [28]. A review of 10 studies led to the conclusion that 5 years after the initial cancer diagnosis, the majority of patients assess the quality of their life as high, especially with respect to relations with others, personal strength, spiritual change, and appreciation of life. Additionally, one analysis showed that 10 years after recovery patients enjoyed greater satisfaction with life and better personal relations as compared with the control group [23].

The above-cited examples from the literature indicate that PTG researchers have tried to explain the phenomenon using various contexts, considering such factors as culture, type of cancer, duration, depressive symptoms, impact of active coping, and catering for one’s own needs. Overall, it can be concluded that PTG is strongly positively linked to the occurrence of traumatic experiences, in particular, oncological breast disease. Our study corroborates the results to date. For example, it emerged that our respondents perceived the greatest changes in terms of appreciation of life, with lesser changes in their appreciation of relating with others, self-perception, and spiritual change.

Psychological theory interprets spirituality as an aptitude for spiritual experiences manifested in the transcendent sphere and moral sensitivity, as well as coping in ultimate situations. These are personal resources that can be used to deal adaptively with problems, especially difficult and traumatic problems [29]. It was assumed that a traumatic experience associated with breast cancer leads to deeper changes in the spiritual sphere. Current studies have shown that spirituality was positively correlated with PTG in breast cancer survivors. A systematic review of 47 papers demonstrated that 75% of breast cancer patients manifested PTS symptoms and PTG, the latter interpreted as an increase in personal strength, relationships, and appreciation of life [23]. The results presented in 23 publications from 2002 to 2016 confirmed the positive impact of spiritual growth on PTG in cancer-diagnosed and treated patients [28]. Psychologically, spirituality can be viewed from two perspectives. It can be understood as religiousness or something distinct from religion. Spirituality is defined in the literature as potential adaptive resources which an individual utilises in the face of tragic events. Their role is to help a person to cope with adversity, to protect them from the destructive influence of events, and as a result, to initiate PTG [30].

Studies conducted in the USA using the Eastern Oncology Group database intended to verify the following hypotheses: (1) Spirituality will be negatively linked to emotional distress in patients, (2) spirituality will have a positive influence on PTG, and (3) the spirituality of the patients and their partners affects each other. The results confirmed the first two assumptions. However, the partner’s spirituality had no bearing on PTG [28].

In an analysis carried out in 2019 in Croatia, it was established that respondents with PTG (72.5% of the participants experienced moderate positive changes) reported very slight changes in the spiritual sphere [26].

Various studies have confirmed that women with breast cancer experience PTG in the realm of personal relations, spirituality, and appreciation of life. The increased manifestation of these values in the process of adapting oneself to illness can counteract the negative consequences of disease [25]. The women affiliated with Polish Amazon Clubs whom we interviewed manifested spiritual changes at a rather low level (2.95 ± 1.33). A lack of significant growth in the spiritual sphere could have been due, for example, to high functioning of respondents before their illness, which was difficult to assess in the present study. They were not asked how they assessed their spiritual growth; they were only asked about the fact of growth after the cancer experience. Furthermore, the scope of analysis was limited to individuals belonging to the helping association, which may have affected the study results. The breast cancer survivors received social support, and they were not in urgent need of seeking spiritual growth. Further research should explore differences in various approaches to this issue, comparing them with results obtained from studies involving non-members of Amazon Clubs.

American investigators have found that high PTG accompanied younger non-white women who underwent chemotherapy. While going through greater difficulties related to the course of their illness, they experienced depressive states and manifested active coping, unlike patients with lower PTG. Older women manifested lower PTG. Similarly, a lower level of depressive symptoms and active, adaptive coping was noted; the diagnosis was not as disruptive to them as the younger group [31]. Most studies have not confirmed the impact of age, marital status, or income on PTG [32], although there are also studies that have demonstrated a positive relationship between older age and PTG. It was noticed in victims of sexual assault and those with an acquired brain trauma [33,34]. In a group of breast cancer patients in Slovenia, age did not increase differences in PTG values [35].

### 4.3. Relationship of PTG with Resilience

Resilience and PTG should be viewed as two distinct concepts; however, despite the differences, they are closely interlinked. The present research seems to confirm this by establishing the co-occurrence of PTG and resilience (*r* = 0.54, *p* < 0.05). This suggests there is a possibility of optimising breast cancer care through activities intended to strengthen resilience. In this way, the negative impact of physical, psychological, and social changes occurring during treatment can be mitigated, and health-promoting behaviours can be developed. Our results were consistent with the conclusions of other studies, which havee suggested that resilience increased the likelihood of PTG in both HIV-positive individuals [36] and women with infertility [37].

An earlier study of women with cancer showed that the trauma of breast cancer contributed to positive changes in their lives. Resilience significantly correlated with the majority of PTG factors. Highly resilient women showed more positive changes, especially in their self-perception and appreciation of life. However, no correlation was observed between the level of resilience and changes in relating with other people or in the spiritual sphere. The study involved only women residing in Lodz Voivodeship, who underwent mastectomy during their treatment. The present study also demonstrates the co-occurrence of PTG and resilience, mainly in terms of personal resilience, which aligns with earlier conclusions. Further, changes in social competences were also noticed, unlike in the analysis mentioned above. This difference may be due to the more diverse study group. For example, women whose treatment plan did not include breast removal were not excluded from our survey, and the respondents were from various regions of Poland [9].

A systematic review of 39 studies established that resilience was related to clinical, sociodemographic, social, and psychological variables, with the latter having the greatest significance, and that psychological stress was most commonly associated with reduced resilience. The majority of the studies relied on various forms of the Connor–Davidson Resilience Scale (CD-RISC) (*n* = 20) [38].

In a China-based cross-sectional study involving 260 women with breast cancer, it was observed that resilience was significantly correlated with anxiety, depression, and PTG. All participants underwent chemotherapy. However, one exclusion criterion was a recent experience of another severe traumatic event, existing mental disease, or a serious concurrent illness. To obtain data, the study utilised the PTGI, the Hospital Anxiety and Depression Scale (HADS, a Chinese hospital scale revised in line with scales used abroad), and the CD-RISC scale. Fear and depression both correlated negatively with resilience and PTG, but there was a significant positive correlation between resilience and PTG. It was shown that resilience had a direct positive influence on PTG, Additionally, it mitigated anxiety and depression during chemotherapy. This suggests the need for psychological rehabilitation available in oncological wards, as this should promote PTG [39].

There are also studies that havee not confirmd positive correlations between PTG and resilience. Such observations were made in Israel and Lebanon, where the PTG result obtained in highly resilient people was rather low. These findings may be due to the fact that people with high levels of resilience are less likely to experience psychiatric disorders including PTSD [40].

In the present study, no statistically significant differences in PTG (nor its components) were found in our respondents. Here, we surveyed women aged 31 and older, which was dictated by the nature of the disease. In the case of other cancers typical of other age groups, the results could be different. We do not know the reaction of very young women to cancer, and this, surely, would be very difficult to ascertain. It is possible that we would have obtained better scores for older persons if we had surveyed a different traumatic event. This may encourage comparative studies in this respect.

Resilience was been shown to play a mediating role between a coping strategy in a difficult situation and the quality of relating with others. The higher the level of the partners’ planning in a difficult situation, the higher the level of their resilience, thus, increasing their commitment to the relationship [41]. As such, resilience has a positive effect on the creation and maintenance of satisfying social bonds [41]. In light of the above, it was assumed that it would be possible to observe different levels of PTG in married and unmarried women. However, no statistically significant differences were found in this area (*p* > 0.05). Future research in this area would have to include those in informal relationships as well, which would make it possible to determine the overall impact of the romantic relationship and partner support on the ability to cope with traumatic events and PTG.

Research carried out in the USA using the PTGI within the first two years of a breast cancer diagnosis has indicated that women experiencing moderate to high growth had previously had high levels of intrusive thoughts about the problem or they suffered depressive symptoms. In contrast, those with low PTG were less depressed and not affected by high invasiveness of their illness. The results also suggest that a high level of active and adaptive coping favours high PTG [35].

Our study showed that women with additional traumatic experiences had higher PTG (74.46 ± 13.30) than those facing breast cancer alone (80.60 ± 12.98). Thirty-five out of 100 respondents mentioned various other personal tragedies, typically one or two events. The assumption that additional problems decrease growth was not confirmed; the reason for this was not discovered. It may be due to the fact that all respondents received support in Amazon Clubs. There have been no similar studies conducted among persons not affiliated with such an organisation. A more detailed study could determine to what degree the accumulation of dramatic experiences causes growth and when it leads to decreased growth. The present results clearly show that patients with one traumatic experience (breast cancer) manifested lower positive changes in self-perception and relations with others as compared with patients struggling with additional traumatic events. Changes in appreciation of life and spirituality were not statistically significant.

## 5. Conclusions

Our research has demonstrated that the trauma presented by breast cancer is conducive to positive changes in the life of the patients. These changes are noticeable mainly in appreciation of life, relations with others, and self-perception, and less in the spiritual sphere. This implies that such women become more aware, self-confident, and more committed to their loved ones. Despite such benefits, the results we obtained also indicated the co-occurrence of negative effects. Three quarters of the interviewed women presented symptoms indicative of PTSD, related mainly to intrusion, arousal, and avoidance. This may suggest that persistent thoughts of the trauma encourage its processing, resulting in a greater likelihood of developing PTG. Due to such experiences, women should better cope with other stressful situations in the future.

Roughly, one-third of respondents (35%) reported the presence of traumatic events other than cancer. It was in this group that higher PTG was observed relative to those for whom breast cancer was the only trauma at the time. In addition, the proportion of respondents who were exposed to more stressful events manifested higher levels of changes in self-perception and changes in relationships.

The results described here confirm the co-occurrence of post-traumatic development and resilience, especially personal resilience and social competence. The ability to benefit from trauma is a sign of good adaptation and mental health, thus, preventing various mental dysfunctions and protecting people against some negative effects such as PTSD.

The level of the observed PTG was affected neither by age nor marital status. The present study, however, cannot unambiguously rule out such a relationship due to the relatively low diversity of the respondent group. Almost half of the respondents (46%) were women aged 61–70 years, and most of them (60%) were married at the time of the survey. In order to analyse this relationship more reliably, it would be necessary to reach out to more people below the age of 40, as this age group was a distinct minority (15%).

This study, due to a small number of respondents and its cross-sectional character, did not allow the direction of the correlation to be unambiguously determined. Nonetheless, it points to the need for further research in this area.

## Figures and Tables

**Table 1:** Study group description (*n* = 100).

Variable	Class	*n*	Cum. No.	%	Cum. %
Age	≤40 years	15	15	15	15
	51–60	18	33	18	33
	61–70	46	79	46	79
	71–80	21	100	21	100
Time	<5	22	22	22	22
	5–10	18	40	18	40
	>10	60	100	60	100
Place of residence	Town	91	91	91	91
	Countryside	9	100	9	100
Education	Primary or vocational	18	18	18	18
	Secondary	33	51	33	51
	Higher	49	100	49	100
Marital status	Married	60	60	60	60
	Single	5	65	5	65
	Widow	21	86	21	86
	Cohabiting	6	92	6	92
	Divorced or separated	8	100	8	100
Other traumatic events	Yes	35	35	35	35
	No	65	100	65	100
PTSD	Yes	77	77	77	77
	No	23	100	77	100

Time—time from the diagnosis of the disease; Cum. No.—cumulative numbers; Cum. %—cumulative %.

**Table 2:** Descriptive statistics of quantitative variables.

		Mean	*SD*	Min.	Max.
PTGI	Post-traumatic growth	76.61	13.45	38.00	104.00
	Self-perception	3.60	0.73	1.22	4.89
	Relations	3.73			
	Appreciation of life	4.00	0.86	1.33	5.00
	Spiritual changes	2.95	1.33	0.00	5.00
IES-R	Post-traumatic stress symptoms	49.57	16.08	6.00	81.00
	Intrusion	2.33	0.85	0.00	4.00
	Arousal	2.22	0.79	0.00	3.88
	Avoidance	2.19	0.72	0.00	3.50
KOP-26	Resilience	103.80	16.57	58.00	129.00
	Personal competences	3.39	6.29	19.00	45.00
	Family competences	46.93	7.46	24.00	55.00
	Social competences	21.48	4.99	9.00	30.00
Significance support of	Family	4.37	0.81	2.00	5.00
	Others	4.00	0.83	2.00	5.00
	Medical staff	4.02	0.84	1.00	5.00
	Other sick persons	4.25	0.66	3.00	5.00

PTGI—Post-traumatic Growth Inventory, IES-R—Impact of Event Scale–Revised; KOP-26—Resilience Assessment Questionnaire.

**Table 3:** Correlation matrix.

	Age	Time	PTG	Res.	PComp	FComp	SComp	PTSS	Intr	Arous
Time	0.60 *	x								
PTG	−0.06	0.03	x							
Resilience	−0.11	−0.13	0.54 *	x						
PComp	−0.14	−0.16	0.54 *	0.88 *	x					
FComp	−0.07	−0.10	0.38 *	0.88 *	0.69 *	x				
SComp	−0.02	−0.02	0.45 *	0.84 *	0.64 *	0.62 *	x			
PTSS	−0.01	−0.14	0.30 *	0.16	0.21 *	0.15	0.00	x		
Intr	0.02	−0.07	0.25 *	0.10	0.16	0.15	−0.08	0.91 *	x	
Arous	−0.07	−0.19	0.31 *	0.15	0.17	0.11	0.05	0.95 *	0.81 *	x
Avoid	0.08	−0.09	0.28 *	0.22 *	0.29 *	0.18	0.06	0.82 *	0.63 *	0.71 *

Time—time from the diagnosis of the disease; PTG—post-traumatic growth; PComp—personal competences; FComp—family competences; SComp—social competences; PTSS—post-traumatic stress symptoms; Intr—Intrusion; Arous—arousal; Avoid—avoidance; *—*p* < 0.05.

**Table 4:** Differences in post-traumatic growth and its components in women of different ages (Kruskal–Wallis test).

Age (Years)	31–50	51–60	61–70	71–80	H	*p*
	*n* = 15	*n* = 18	*n* = 46	*n* = 21		
PTG, *M* (*SD*)	77.33 (15.61)	79.89 (15.97)	74.76 (11.55)	77.33 (13.74)	2.63	0.45
Self-perception, *M* (*SD*)	3.62 (0.95)	3.77 (0.86)	3.53 (0.65)	3.60 (0.62)	1.95	0.58
Relations with others, *M* (*SD*)	3.78 (0.60)	3.87 (0.81)	3.57 (0.58)	3.91 (0.57)	5.96	0.11
Appreciation of life, *M* (*SD*)	4.24 (0.60)	4.22 (0.85)	3.99 (0.85)	3.67 (1.01)	4.78	0.19
Spiritual changes, *M* (*SD*)	2.80 (1.51)	3.00 (1.25)	2.87 (1.16)	3.17 (1.63)	1.90	0.59

PTG—post-traumatic growth.

**Table 5:** Differences in post-traumatic growth and its components in women with illnesses of different durations (Kruskal–Wallis test).

Time (Years)	<5	5–10	>10	H	*p*
	*n* = 22	*n* = 18	*n* = 60		
PTG, *M* (*SD*)	77.41 (13.46)	74.17 (13.12)	77.05 (13.68)	1.37	0.51
Self-perception, *M* (*SD*)	3.63 (0.80)	3.59 (0.66)	3.60 (0.73)	0.21	0.90
Relations with others, *M* (*SD*)	3.76 (0.62)	3.51 (0.79)	3.78 (0.62)	2.64	0.27
Appreciation of life, *M* (*SD*)	4.22 (0.61)	3.93 (0.92)	3.94 (0.92)	1.03	0.60
Spiritual changes, *M* (*SD*)	2.86 (1.42)	2.64 (1.26)	3.07 (1.31)	2.08	0.35

PTG—post-traumatic growth.

**Table 6:** Differences in post-traumatic growth and its components in married and unmarried women (Mann–Whitney U test).

	Married Women	Unmarried Women	Z	*p*
	*n* = 60	*n* = 40		
PTG, *M* (*SD*)	78.33 (11.23)	74.03 (16.03)	0.99	0.32
Self-perception, *M* (*SD*)	3.69 (0.59)	3.46 (0.89)	0.92	0.35
Relations with others, *M* (*SD*)	3.76 (0.60)	3.69 (0.72)	0.28	0.78
Appreciation of life, *M* (*SD*)	4.08 (0.77)	3.89 (0.99)	0.58	0.56
Spiritual changes, *M* (*SD*)	3.17 (1.45)	2.61 (1.51)	1.84	0.06

PTG—post-traumatic growth.

**Table 7:** Differences in post-traumatic growth and its components in women experiencing one and many traumatic events (Mann–Whitney U test).

	One Traumatic Event: Cancer, *n = 65*	>1 Traumatic Event, *n* = 35	Z	*p*
PTG	74.46 (13.30)	80.60 (12.98)	2.38	0.02
Self-perception	3.48 (0.75)	3.83 (0.64)	2.19	0.03
Relations with others	3.65 (0.63)	3.88 (0.67)	1.97	<0.05
Appreciation of life	3.94 (0.89)	4.12 (0.82)	1.04	0.29
Spiritual changes	2.79 (1.37)	3.24 (1.20)	1.55	0.17

PTG—post-traumatic growth.

## Data Availability

The data presented in this study are available on request from the corresponding author.
